# Obstetric outcomes and effects on babies born to women treated for epilepsy during pregnancy in a resource limited setting: a comparative cohort study

**DOI:** 10.1186/s12884-018-1857-3

**Published:** 2018-06-14

**Authors:** Priyadarshani Galappatthy, Chiranthi Kongala Liyanage, Marianne Nishani Lucas, Dilini T. L. M. Jayasekara, Sachith Aloka Abhayaratna, Chamari Weeraratne, Kusum De Abrew, Padma Sriyani Gunaratne, Ranjani Gamage, Chandrika N. Wijeyaratne

**Affiliations:** 1 Department of Pharmacology, Faculty of Medicine, University of Colombo, Colombo, Sri Lanka; 2 Department of Peadiatrics, Faculty of Medicine, University of Colombo, Colombo, Sri Lanka; 3 National Hospital of Sri Lanka, Colombo, Sri Lanka; 4 Department of Obstetrics and Gynecology, Faculty of Medicine, University of Colombo, Colombo, Sri Lanka

**Keywords:** Women with epilepsy, Pregnancy, Carbamazepine, Antiepileptic drugs, Perinatal outcome, Obstetric outcome

## Abstract

**Background:**

Management of epilepsy during pregnancy in a resource-limited setting (RLS) is challenging. This study aimed to assess obstetric outcomes and effects on babies of women with epilepsy (WWE) exposed to Anti-epileptic drugs (AEDs) compared to non-exposed controls in a RLS.

**Methods:**

Pregnant WWE were recruited from antenatal and neurology clinics of a tertiary care hospitals in Sri Lanka. Patients were reviewed in each trimester and post-partum. Medication adherence, adverse effects, seizure control and carbamazepine blood levels were monitored. Post-partum, measurements for anthropometric and dysmorphic features of the babies and congenital abnormalities were recorded. Age and sex matched babies not exposed to AED recruited as controls were also examined.

**Results:**

Ninety-six pregnant WWE were recruited (mean period of gestation 22.9 weeks). Mean age was 28 years and 48(50%) were primigravidae. Fifty percent (48) were on monotherapy, while 23.8, 15.9 and 4.1% were on two, three and four AEDs respectively. AEDs in first trimester (TM1) were carbamazepine (71%), valproate (25.8%) clobazam (29.5%), lamotrigine (7%) topiramate (5%) and others (3.4%). Sodium valproate use reduced significantly from T1 to T2(*p* < 0.05). Sub-therapeutic carbamazepine levels correlated positively (*r* = 0.547) with poor medication adherence (*p* = 0.009) and negatively (*r* = 0.306) with adverse effects (*p* = 0.002). Seventy-six WWE completed follow-up reporting w 75 (98.6%) live births and one T1 miscarriage (1.3%). Three (4.3%) were preterm. Majority (73.33%) were normal vaginal deliveries. Cesarean sections were not increased in WWE. Fifty-nine (61.45%) babies were examined. For those examined during infancy, 53 age and sex matched controls were recruited and examined.. Congenital abnormalities occurred in 5 (9.43%) babies of WWE [atrio-ventricular septal defect (2), renal hypoplasia (1), cryptorchidism (1), microcephaly (1)] compared to 2 (3.77%) in controls (2 microcephaly; *p* = 0.24). Fetal exposure to AEDs increased a risk of low birth weight (RR 2.8; *p* = 0.049). Anthropometric parameters of AED exposed babies were lower at birth but not statistically significant between the two groups (weight *p* = 0.263, length *p* = 0.363, occipito-frontal circumference (OFC) *p* = 0.307). However, weight (*p* = 0.009), length (*p* = 0.016) and OFC (*p* = 0.002) were significantly lower compared to controls at an average of 3.52 months.

**Conclusion:**

Most pregnancies are unplanned in the RLS studied, and AEDs were altered during pregnancy. Congenital anomalies occurred at rates comparable to previous reports. Fetal exposure to AED had growth retardation in infancy compared to non-exposed babies.

## Background

Epilepsy is the commonest neurological condition with an impact on outcome of pregnancy affecting 3 to 4 per 1000 pregnancies [1]. It has an age-adjusted incidence of 16 to 51 per 100,000 [2] in the general population. Although use of antiepileptic drugs during pregnancy is associated with major congenital malformations in the fetus, they cannot be discontinued in many women planning pregnancy because of the risk of uncontrolled seizures that can be harmful to the mother as well as to the child [3, 4]. Therefore, children of women with epilepsy (WWE) are at a two to three times higher risk of congenital malformations compared with the general population [5, 6]. The highest risk of malformations is reported with sodium valproate (VPA) with patients on higher doses and polytherapy being at a greater risk than those on monotherapy [5, 7]. In addition to the risk of structural malformations, there is increasing concern regarding adverse effects of AEDs on cognitive development of babies born to mothers with epilepsy [8]. Furthermore, a follow-up study at 6 years has shown that fetal exposure to VPA is associated with reduced cognitive abilities across multiple domains [9]. However, there are sparse data on the effect of AEDs during pregnancy in Resource-Limited Settings (RLS) from Lower and Middle-Income Countries (LMIC). There is also less data on the physical growth of babies born to mothers with epilepsy. This prospective comparative cohort study was conducted to determine maternal and obstetric outcomes in the mother due to AED and the effects on babies born to women with epilepsy treated with AEDs during pregnancy in Sri Lanka.

## Methods

### Study design, setting and patient selection

A prospective comparative cohort study was conducted in WWE who received AEDs during pregnancy over a period of 3 years and 5 months from September 2011 through February 2015. Pregnant women on single as well as multiple AEDs attending antenatal clinics of the De Soyza Maternity Hospital (DMH) and neurology clinics of the National Hospital of Sri Lanka (NHSL) who consented to participate were included. Selected facilities were the main tertiary care referral centers in Colombo, Sri Lanka for such patients. Those having a history of any genetic disorder were excluded. Sample size was calculated using the reported relative risk (RR) of 5 for occurrence of congenital malformations with carbamazepine or valproate, compared to the malformation rate in pregnant women without risk factors [3]. Sample size of 47 per each group was required to see congenital malformations, in WWE, with a 95% confidence interval and 80% power to observe the difference. Considering the number of eligible pregnant WWE seen at the clinics, total of 94 patients were targeted for recruitment allowing for dropouts. Information on basic demographics, maternal obstetric and medical history, comorbidities, exposure to known teratogens (i.e. alcohol, cigarette smoking) and current medications were obtained through a structured interviewer administered questionnaire. Mothers’ medical and obstetric records were used to confirm the information. Study participants were reviewed during first, second and third trimester antenatal clinic visits and once after the delivery. During review visits, seizure control, patient adherence to treatment (via interviewer administered medication adherence scale), any side effects of AEDs experienced and obstetric condition was recorded. Details of the delivery and anthropometric data of the baby at birth were obtained from maternal medical records and child health and development records following delivery.

### Therapeutic drug monitoring

Monitoring serum AED levels is not routinely available even during pregnancy in Sri Lanka. However, for the purpose of the study serum carbamazepine level was measured once during each trimester and in the postpartum visit as most patients were on carbamazepine. Serum carbamazepine levels were measured using a Latex Enhanced Immunoterbidimetric (LEI) method in Randox clinical chemistry analyzer. There were no facilities to monitor serum levels of other AEDs.

### Control group

Babies for the control group were recruited from the maternity hospital and an immunization clinic in Colombo. As the babies of WWE were delivered at different local hospitals, babies were examined at different time points when the mothers attended neurology/post-natal clinics post-partum. Babies not exposed to AED and have not suffered any perinatal insults matched for age of examination (± 1 month) and sexes of the babies born to WWE were recruited.

### Definitions used

**Fig. 1:**
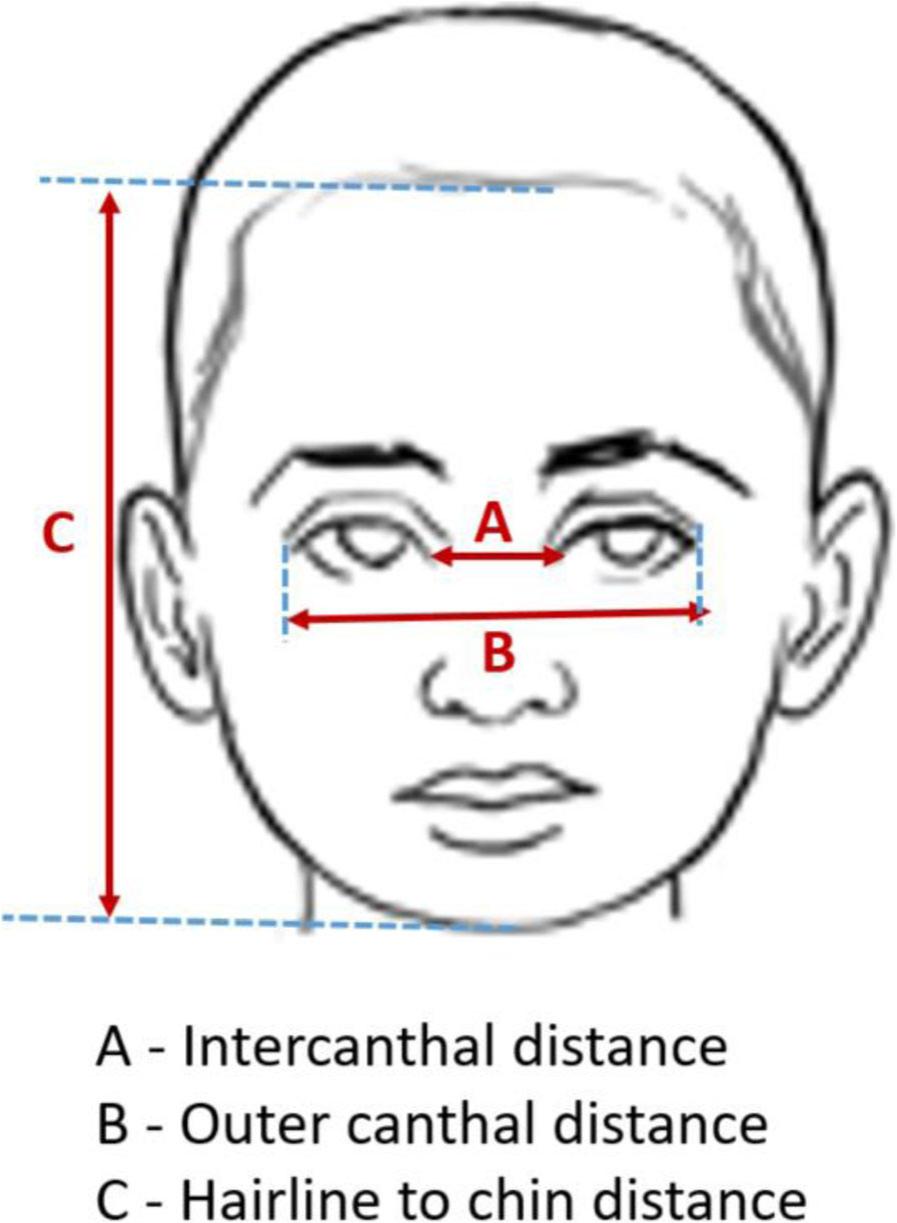
Facial measurements used to ascertain the presence facial dimorphism

Intrauterine growth retardation was defined as fetal weight that is below the 10th percentile for gestational age as determined through an ultrasound by the attending consultant obstetrician. Preterm delivery was defined as birth occurring before 37 weeks of gestation. Low birth weight was defined as birth weight of 2.499 Kg or less in a baby regardless of gestational age. Congenital anomalies were defined as structural defects, chromosomal abnormalities, inborn errors of metabolism, and hereditary disease diagnosed before, at, or after birth [10]. Facial dysmorphisms were defined as mid-facial hypoplasia, hypertelorism and shallow philtrum, which were determined from the measurements made shown in Fig. 1. Mid-facial hypoplasia also known as midface retrusion was defined as posterior positioning and/or vertical shortening of the infraorbital and perialar regions, or increased concavity of the face and/or reduced nasolabial angle [11]. Philtrum smoothness was measured according to the 5-point Likert scale used in the pictorial Lip- Philtrum Guide described by Astley and Clarren [12] A score of 5 represents the flattest philtrum and thinnest vermilion border of the upper lip whereas a score of 1 represents the most prominent philtral columns and most defined upper lip [12]. Babies who had Astley Clarren score of 4–5 were considered to have smooth philtrum. The term hypertelorism means an increased distance between two body parts whereas “ocular hypertelorism” indicates significantly widely placed eyes [13]. Telecanthus means increased inner canthal distance without significant lateral displacement of the lateral orbital walls [13]. Microcephaly was defined as a reduction in the size of the brain with a skull circumference more than 3 SD below the mean for sex, age and ethnic origin [14].

### Outcomes evaluated

Primary outcomes were structural congenital malformations which had clinical manifestations, facial dysmorphisms in the form of mid facial hypoplasia, hypertelorism, smooth philtrum and anthropometric parameters in the two groups as determined by detailed examination of babies with relevant measurements taken for anthropometric and facial dimorphic features. All babies of WWE were examined by a single consultant neonatologist. Babies in the control group were examined by trained medical doctors under the supervision of a single consultant neonatologist. If there were any doubts on any of the parameters evaluated, they were cross-checked by the consultant neonatologist. The congenital abnormalities that were detected were confirmed through investigations where relevant. The babies selected for the comparative sample were also examined for the same parameters by the same team.

The secondary outcomes evaluated were maternal seizure control, side effects of therapy in the mother, adherence to therapy and serum carbamazepine levels, live births, preterm deliveries and complications during the delivery.

Anthropometric parameters at birth were obtained from the Child Health Developmental Record, whereas weight, length and occipito-frontal circumferences were measured according to the WHO guidelines during the follow up visits [15].

The Ethics Review Committee of the Faculty of Medicine, University of Colombo, Sri Lanka, and the institutional ethics review committees of the two recruitment centers approved the study. Furthermore, permission was obtained from the Medical Officers of Health in-charge of the immunization clinics for recruitment of the control group. Informed written consent was obtained from all the pregnant women included in the study at the point of recruitment and from the mothers of the controls.

### Statistical analysis

The anthropometric parameters and other adverse outcomes of babies exposed to AEDs were compared with that of the control group. Dichotomized variables were analyzed by Pearson’s chi-square test. Fisher’s exact test was used for cross-tabulated measures for small samples with expected cell count < 5. Eta was used to assess directional measures for interval variables (e.g. number of maternal seizures during epilepsy, number of AEDs received in first trimester). Arithmetic means calculated for weight, length, and OFC at birth and at follow up were compared using the paired sample t test. *P*-values < 0.05 were considered statistically significant. Age of the mother, smoking during pregnancy (yes/no), prior pregnancy birth defects and complications (yes/no), complications during the delivery, birth order (first, second, third or more) of the child, adherence to AED therapy, and other medications used were other covariates examined. Statistical analyses was performed using SPSS 17.0 for Windows (SPSS Inc., Chicago, IL, U.S.).

## Results

**Fig. 2:**
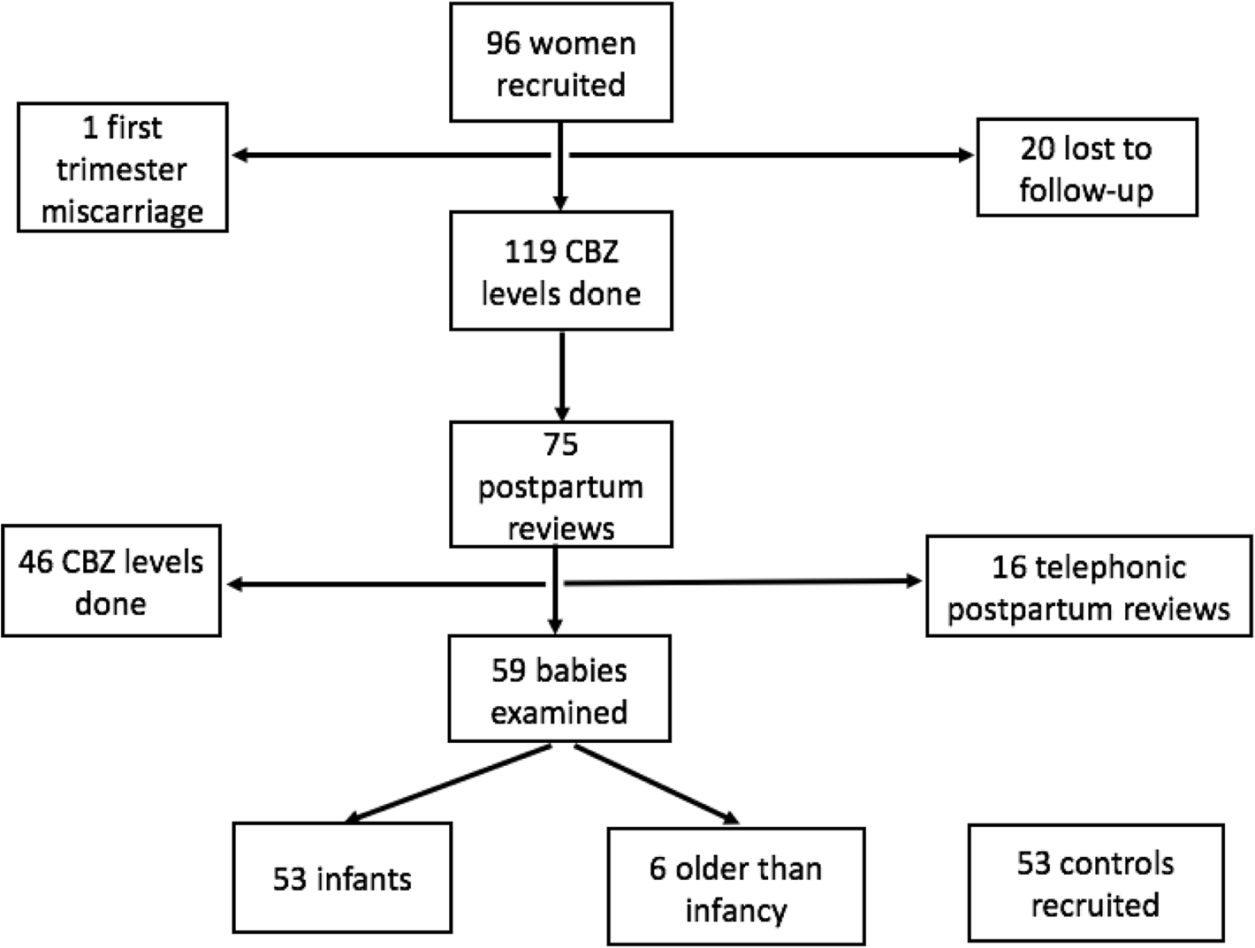
Follow-up of study participants

Ninety-six pregnant women on antiepileptic medication, with an average age of 28 years and a mean period of gestation of 22.9 (± 9.4) weeks were recruited from antenatal and neurology clinics of the two hospitals. Figure 2 illustrates the follow-up of these participants. Fifty percent (*n* = 48) were primigravidae. The characteristics of the recruited pregnant women are summarized in Table 1.

**Table 1:** Characteristics of the pregnant women recruited in to the study

	Study group (*n* = 96)		Controls *n* = 53
	Mean (SD)	Range	Mean (SD)
Age of the mother (years)	28 (5.32)	18–38	28.9 (6.3)
POG at recruitment (weeks)	22.45 (10.0)	6–38	–
Parity (mode)	1	1–5	1
Consanguinity (n, %)	2 (2.0)	–	5 (9.4)
Duration of epilepsy (years)	15.21 (8.86)	0–35	–
Number of antiepileptic drugs used	1.64	1–4	–
Exposure to teratogens			
Smoking	0		0
Passive Smoking (%, n)	9.3 (9)		3.77 (2)
Alcohol (%, n)	1 (1)		0 (0)
Comorbidities (%, n)			
Diabetes & GDM	4.1 (4)		5 (9.61)
Hypertension & PIH	4.1 (4)		–
Valvular heart disease	6.2 (6)		–
Bronchial asthma	9.3 (9)		–
Hypothyroidism	2 (2)		–
Systemic lupus erythematosus	1 (1)		–
Others^a^	5 (5.2)		1 (1.93)

*POG* Period of Gestation, *GDM* Gestational Diabetes Mellitus, *PIH* Pregnancy Induced Hypertension

^a^psychiatric illnesses, beta thalassemia, migraine, anemia, urinary tract infection

### AED therapy

**Fig. 3:**
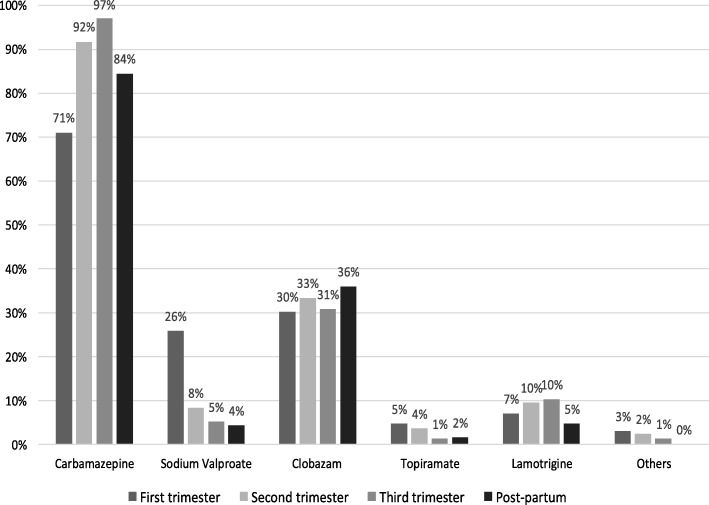
Antiepileptic therapy by trimester and post-partum. The number of women with epilepsy on sodium valproate significantly reduced from first to subsequent trimesters (*P* < 0.05)

Fifty percent (*n* = 48) were on monotherapy while 23.8% (*n* = 21) were on two AEDs, 15.9% (*n* = 14) were on three and 4.1% (*n* = 4) were on four AEDs in the first trimester. There were 22 (25.8%) on sodium valproate in the first trimester (T1) and this number significantly reduced (*p* < 0.05) in subsequent trimesters (T2–8%, T3–5%) while the percentage of mothers on carbamazepine increased considerably from first (71%) to second trimester (91.6%). A summary of AEDs in the study group by trimester is illustrated in Fig. 3. Nine patients (10.22%) have been started on AED after the first trimester.

A total of 165 carbamazepine (CBZ) levels were done with a median of two per study participant. The CBZ levels in each trimester are summarized in Table 2. CBZ levels less than 4 mcg/ml were considered sub-therapeutic, 4–12 mcg/ml were considered therapeutic, and above 12 mcg/ml were considered toxic. Paired sample comparisons in individual patients showed reduction of mean carbamazepine level from T1 to T2 and T2 to T3 (*p* = 0.087) despite a significant carbamazepine dose increase from T2 to T3 (*p* = 0.018).

**Table 2:** CBZ levels by trimester and postpartum

Visit	N	CBZ level in mcg/ml		CBZ level (n, %)		
		Mean (SD)	Range	Sub-therapeutic	Therapeutic	Toxic
PC	3	7.32 (3.58)	3.77–10.93	1 (33.3%)	2 (66.7%)	0
T1	18	6.37 (2.19)	0.50–9.32	2 (11.1%)	16 (88.9%)	0
T2	46	5.57 (2.13)	0.43–9.22	13 (27.7%)	33 (71.7%)	0
T3	52	4.82 (2.38)	0.00–10.16	18 (34.6%)	34 (65.4%)	0
PP	46	5.99 (2.19)	0.11–11.53	6 (13.0%)	40 (87.0%)	0
Total	165	5.25 (2.00)	0.00–11.53	40 (24%)	125 (76%)	0

*PC* preconception, *T1* first trimester, *T2* second trimester, *T3* third trimester, *PP* postpartum

**Fig. 4:**
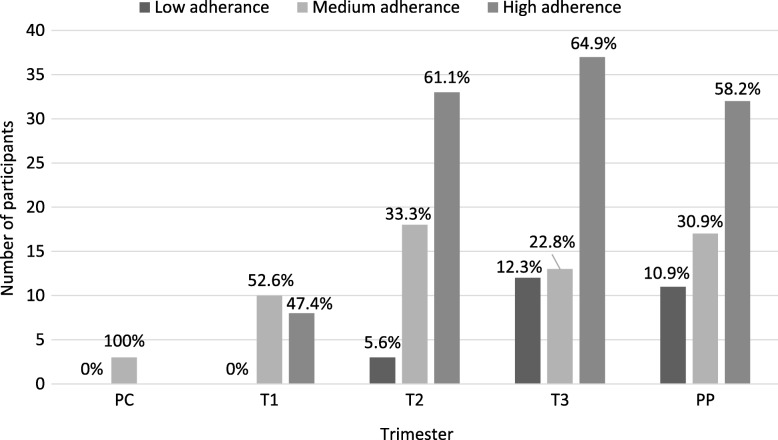
Adherence to antiepileptic drug therapy by trimester and postpartum. The proportion of mothers with high adherence to therapy increased from T1 to T2. However, this was not statistically significant (*p* = 0.28). T1 - first trimester, T2- second trimester, T3- third trimester, PP- postpartum

Most common adverse effects noted were, drowsiness (50.0%), headache (26.0%), dizziness (25.0%), visual disturbances (17.7%), tremor (11.5%), anorexia (9.4%), ataxia (9.4%) and paresthesia (9.4%). Although the proportion of mothers with high adherence to AED therapy increased from T1 (47.4%) to T2 (61.1%), this was not statistically significant (*p = 0.28).* Adherence to AED therapy according to the adherence grades by trimester and postpartum is illustrated in Fig. 4. Low adherence correlated with sub-therapeutic serum carbamazepine levels (*p* = 0.009) and the adherence score correlated negatively with number of adverse effects reported (Pearson’s *R* = − 0.222, *p* = 0.002).

### Maternal outcomes

Total of 76 WWE completed follow up providing data on pregnancy outcomes. There were 75 (98.6%) live-births and one first trimester miscarriage (1.3%). Three of the live births (4.0%) were preterm deliveries. Majority (73.33%) had normal vaginal deliveries (NVD). There was no significant difference in the proportion of preterm deliveries, babies delivered via elective or emergency cesarean sections or assisted vaginal deliveries between the WWE and the control group. Reasons for emergency cesarean sections were uncontrolled seizures (6.66%), poor progression of labor (2.66%), breech presentation (1.33%), placental abruption (1.33%), placenta previa (1.33%), transverse lie (1.33%), premature rupture of membranes (1.33%), and failed induction (1.33%). Majority (64%; *n* = 48) had no seizures during pregnancy while 17.3% (*n* = 13) reported having one seizure and 18.6% (*n* = 14) had two or more seizure episodes during pregnancy. There was no correlation between seizure frequency and change in AEDs during pregnancy, adherence to therapy or measured carbamazepine blood levels.

### Outcome of the babies

Postpartum babies were examined at a mean age of 5.8 months (range 1 day to 30 months). Five (9.43%) congenital abnormalities occurred in WWE exposed to AEDs during pregnancy compared to two (3.77%) in the control group with a relative risk of 2.5 (*p* = 0.24). Moreover, seven (9.2%) had intrauterine growth retardation (IUGR) while 16 (21.33%) had low birth-weight (Table 3). The babies of WWE were more likely to have low birth weight than control group with a relative risk of 2.8 (*p* = 0.049, CI 1.00–7.97). Although the weight, length and OFC at birth were slightly lower in babies exposed to AEDs in-utero, this difference was not statistically significant. However, weight, length and the OFC measurements taken at the follow-up visit of these infants at an average of 5.8 months, were all significantly less than those of babies in the control group (Table 3).

**Table 3:** Adverse pregnancy outcomes and the anthropometric parameters in babies in the control and the study group

		AED group *N* = 75 (%)	Controls *N* = 53 (%)	*P*
Average age of the baby at examination (months) (SD)				
The total study population (*N* = 75)		5.8 (+/− 6.73)		
Cases matched with controls (*N* = 53)		3.52 (2.79)	3.11 (+/−2.28)	0.30
Average POA^a^ at delivery (weeks) (SD)		38.22 (+/−3.50)	38.52 (+/− 2.06)	
Term deliveries		70 (92.1)	48 (90.5)	0.66
Preterm deliveries		5 (6.57)	2 (3.77)	0.49
Sex of the baby				
Male		43 (57.33)	29 (54.71)	0.76
Female		32 (42.66)	24 (45.28)	0.76
Mode of delivery				
NVD^b^		55 (73.33)	34 (64.15)	0.26
Forceps delivery		1 (1.33)	1 (1.88)	0.80
Vacuum delivery		1 (1.33)	3 (5.66)	0.16
Elective LSCS^c^		6 (8)	9 (16.19)	0.06
Emergency LSCS^c^		10 (13.33)	7 (13.20)	0.23
Intrauterine growth retardation (IUGR)		7 (9.33)	1 (1.88)	0.87
Low birth weight		16 (21.33)	4 (−7.54)	*0.03*
Examination findings in babies		*N* = 53	*N* = 53	
Congenital abnormalities (n,%)		5 (9.43)	2 (3.77)	0.24
Atrio-ventricular septal defect		2 (3.77)	0	–
Renal hypoplasia		1 (1.88)	0	–
Cryptorchidism		1 (1.88)	0	–
Microcephaly		1 (1.88)	2 (3.77)	0.35
Anencephaly		0	0	–
Neural tube defects		0	0	–
Cleft palate		0	0	–
Facial measurements (cm)				
Inner-canthal distance		3.02	2.4	0.32
Outer-canthal distance		9.3	8.37	0.83
Hair line to chin distance		12.8	12.15	0.24
Facial dysmorphism				
Mid-facial hypoplasia		1 (1.66%)	2 (3.77%)	0.16
Smooth philtrum (Astley–Clarren scale 4 &5)		16 (28.07%)	1 (1.88%)	*0.0004*
Hypertelorism		1 (1.88%)	1 (1.88%)	0.74
Telecanthus		1 (1.88%)	0	–
Epicanthic folds		1 (1.88%)	0	–
Simian crease		0 (1.88%)	0	–
Anthropometry				
At birth	Weight (Kg)	2.74	2.91	0.263
	Length (cm)	51.52	51.53	0.363
	OFC^d^ (cm)	32.13	32.85	0.307
At the postpartum visit	Weight (Kg)	4.12	4.97	*0.009*
	Length (cm)	55.53	58.09	*0.016*
	OFC^d^ (cm)	37.37	39.49	*0.002*

^a^Period of amenorrhea

^b^Normal vaginal delivery

^c^lower segment cesarean section

^d^Occipito-frontal circumference

Smooth philtrum (Astley-Clarren Scale) and hypertelorism were identified in 28.0 and 1.66% respectively. Babies with smooth philtrum were significantly higher in the AED group (28.07%) compared to the control group (1.88%) with a relative risk of 11.3 (*p* = 0.01, CI 1.5–82.67) (Table 3). Telecanthus was recorded in 1.88% of cases. None of the cases nor controls were found to have any facial measurements that indicate hypoplasia of the mid face, epithanthal folds, short nose, clefts or finger hypoplasia.

Congenital anomalies found in the study group included AV septal defects, renal hypoplasia, microcephaly and cryptorchidism. All these were term babies, born to mothers aged 28 to 34 years. None of these babies had any other features suggestive of a syndrome or genetic disorder. None of these babies were exposed to alcohol or tobacco in-utero. All babies with anomalies were exposed to CBZ. In addition to CBZ, babies who were found to have hypoplastic kidneys, bilateral cryptorchidism and ASD were exposed to lamotrigine, clobazam and both lamotrigine and clobazam respectively. In the two babies who had microcephaly, there was no history of consanguinity.

One first trimester miscarriage was reported in a 34-year-old primigravida who was only on CBZ with good seizure control. The mother did not have a history of recurrent miscarriages or any other co-morbidities. An infant death was reported in a baby, born to a 33-year-old mother who was on CBZ, lamotrigine, and clobazam. This baby had been delivered by an emergency LSCS due to placental abruption and intubated and ventilated due to recurrent seizures. Subsequently the baby had developed a lower respiratory tract infection and sepsis at the age of 4 months and succumbed to the illness. The mother had no other comorbidities and she reported three seizures during the pregnancy. The death was considered unlikely to be related to epilepsy or AED therapy.

## Discussion

Optimal management of women with epilepsy (WWE) during their child bearing years and pregnancy presents a clinical conundrum, even in developed countries as treatment with certain AEDs during pregnancy is associated with an increased risk of major congenital malformations. While possible teratogenicity should be a concern when commencing AEDs in all women of childbearing age, preplanned pregnancies with pre-conceptional folic acid are advised to minimize risk during pregnancy. Avoiding sodium valproate that carries the highest risk of teratogenicity and commencement of minimum effective dose of monotherapy are encouraged pre-conceptionally [16]. Although lamotrigine is considered relatively safe in pregnancy it is not readily available for patients in state hospitals in Sri Lanka [17]. Carbamazepine monotherapy is also considered to have a comparatively lower risk of teratogenicity [18, 19], while sodium valproate carries the highest risk of teratogenicity with 5.4 to 20.3% reported major congenital malformations or death with significantly increased risk at doses more than 600 mg/day. The greatest attributable risk has been observed at doses more 1000 mg/day [20–22].

This study highlights the additional challenges faced by WWE managed in overcrowded busy clinics in RLS during their pregnancies. They include limited patient monitoring by health care professionals, lack of facilities for therapeutic drug monitoring and limited understanding of patients leading to poor planning of pregnancies. Although changing AED is not recommended after conception, in this study there was a significant change in AED therapy from T1 with a reduction of sodium valproate use and an increase in the number on CBZ by T3. This is likely to be due to unplanned pregnancies, and changing AED therapy once the patient is found to be pregnant during T1. However, this has not recorded any untoward outcomes such as increased seizure frequency according to our results. The prevalence of many unplanned pregnancies in WWE, points to a greater emphasis on planned pregnancies as a priority health need in this population and probably in other settings with limited resources. WWE and their partners should receive adequate care and follow-up with peri-conceptional counseling. AED therapy must be adjusted prior to pregnancy to minimize teratogenic effects of AEDs. In countries like Sri Lanka, this process may be hampered by inadequacy of facilities as well as social stigma surrounding epilepsy [23–25]. Nevertheless, majority of WWE in our study group had good seizure control during epilepsy. Monitoring of CBZ levels corroborated well with the low medication adherence, which in turn showed a negative association with adverse effects experienced by WWE. Pregnancy changes the pharmacokinetics of AEDs, with altered absorption, haemodilution, increased clearance, metabolism and protein binding [26–28]. This may lead to poor seizure control. Serial AED level monitoring during each trimester and postpartum helps make appropriate dose adjustments to achieve optimal seizure control at the minimum required dose [27, 29]. In keeping with current literature, we detected a significant drop in CBZ levels from T1 to T3 [30] despite increase in adherence to therapy. Therefore, the drop in CBZ level is likely to be due to changes in pharmacokinetics of AEDs in pregnancy.

Similar to findings from previous studies, most of the WWE in this group delivered via normal vaginal deliveries [31]. Furthermore, we found no significant difference in proportion of WWE who undergo emergency or elective lower segment cesarean sections compared to women without epilepsy. There is conflicting evidence on obstetric complications in WWE in literature. There are some studies that have not found an increased risk of cesarean section in WWE [31–33] while one community based study in Iceland showed that cesarean section was twice more frequent than in the general population [34]. Other obstetric complications in WWE include vaginal bleeding, placental abruption, prematurity and pre-eclampsia [31, 34]. Moreover, the risk of premature labor may be substantially increased in WWE who smoke [35]. However, in our study there was no significant difference in WWE and controls possibly due to small sample size and very low rates of smoking in women in Sri Lanka and low rates of passive smoking also observed in the sample.

Children born to WWE epilepsy are at a higher risk of low birth weight and intrauterine growth restriction (IUGR) [36]. This effect is probably multifactorial, influenced by in-utero exposure to AEDs, genetic factors and environmental factors. The strongest association has been found for CBZ whereas the effect of phenytoin, clonazepam, lamotrigine and gabapentin are thought to be less [37]. In keeping with these findings, we found IUGR and low birth weight was much commoner in children born to WWE, who were predominantly on CBZ, in Sri Lanka. However, in this study, WWE had more comorbid illnesses such as hypertension and valvular heart disease compared to the mothers of the control babies. These may have also contributed the IUGR and low birth weight observed.

In-utero exposure to AEDs has been found to adversely affect neurocognitive development in offspring of WWE [8, 9, 38–42]. Children born to WWE have impaired gross motor skills, fine motor skills, language skills and personal-social skills. Although effects of in-utero exposure to AEDs on cognitive development have been studied extensively through follow-up studies, data on physical growth and development appear spares. We found that children born to WWE had significantly lower weight, length and OFC compared to controls by a case by case analysis of anthropometric parameters during a follow up visit (average at 6 months). Similar findings have been found even 20 years after birth, where offspring of WWE have had lower height, weight and body mass index (BMI) compared to those born to women without epilepsy [43]. Compromised growth of babies of WWE could be due to a multitude of factors, including presence of AEDs in breast milk, refraining from or early discontinuation of breast-feeding to avoid AED exposure in the baby, lack of stimulation due to adverse effects of AEDs on the mother and maternal socioeconomic factors. Contrary to the common belief, continuous breastfeeding in these children is associated with less impaired development at ages 6 and 18 months compared to those who are not breastfed or discontinued early [44]. Therefore, further studies are needed to identify and delineate such factors that might affect physical growth and neurocognitive development of children who are exposed to AEDs in-utero.

Many previous studies have confirmed that in-utero exposure to AEDs increases the risk of major congenital malformations (MCMs) with the severity and the nature MCMs being related to the type, dose and number of AEDs [3, 7, 19, 45–47]. The risk of MCMs with carbamazepine, oxcarbazepine, or phenytoin is reported to be less [7] compared to sodium valproate [31, 45]. Similar to previous studies [48], we also found a two to three fold increase in the risk of congenital malformations in babies born to WWE who are exposed to AEDs in-utero compared to unexposed controls, although our findings were not statistically significant probably due to small sample size. Our rates on congenital malformations in both exposed and unexposed groups, were comparable to data from developed countries, probably reflecting the lack of significant effect of other factors operating in RLS settings contributing to congenital malformations. All, except one baby with congenital malformations in this group were exposed to more than one AED in-utero. Cardiac malformations occurring in CBZ monotherapy is well recognized [49, 50], and this was supported by atrial septal defect (ASD), we recorded in a baby exposed only to CBZ. Although we report a case of bilateral undescended testes in a baby exposed to CBZ and clobazam, a meta-analysis in 2017 had concluded that undescended testes was not associated with in-utero exposure to AED [51]. Renal malformations do occur with in-utero exposure to AED and unilateral or bilateral multicystic dysplastic kidneys (MCDK) have been reported in babies exposed to CBZ and phenobarbital [52]. Nevertheless, there were no reports of renal hypoplasia associated with in-utero exposure to AEDs. Smooth philtrum, which is well recognized in fetal alcohol syndrome, has been reported with in-utero exposure to valproic acid [50] and it was significantly more in babies of WWE. However, we were unable to identify significant proportion having any other facial dysmorphic features such as telecanthus and hypertelorism, despite doing specific measurements to identify these, probably due to small sample size of babies examined.

We acknowledge the limitations of this study, which was biased towards difficult-to-manage WWE with other co-morbidities recruited and followed up from antenatal clinics in a tertiary care center in a RLS. There was a high number of patients who were lost to follow up, despite taking all efforts to trace patients, due to limited resources available to the study team and limited contact facilities of patients coming from different parts of the country. Moreover, as the control group of babies were recruited as age and sex matched controls after completing post-partum reviews of the cases, they were recruited postpartum and examined at different ages. Therefore, there was no longitudinal follow-up of the mothers of the control group babies during pregnancy. We were not able to delineate contributory factors for adverse obstetric and children’s’ outcomes, due to the small numbers of study subjects, which prevented obtaining statistically significant results for these parameters. Absence of data on folic acid use, breast-feeding, weaning practices and parental anthropometry are also limitations in this study.

## Conclusion

Our data highlight the challenges faced by WWE during pregnancy in a RLS, due to poor planning of pregnancies and limited monitoring, including lack of therapeutic drug monitoring. A significant proportion of pregnancies in WWE in the study were unplanned and AEDs were altered during the pregnancy. In-utero exposure to AED increased the risk of congenital malformations by two to three-fold similar to reported rates from studies done in other settings. Majority of WWE deliver via normal vaginal deliveries and the risk of cesarean sections is not higher in WWE in our setting. However, risk of low birth weight was greater in babies born to WWE and anthropometric parameters were comparatively lower in the babies exposed to AED in utero. We also noted reduced physical growth during infancy indicated by lower weight, length and OFC of babies exposed to AEDs in-utero than non-exposed babies. Further larger studies with long-term follow-up during post-partum period are needed in RLS to identify reasons for growth retardation in babies exposed to AEDs in-utero and in post-partum period.

## Abbreviations

AED: Antiepileptic drug
ASD: Atrial septal defect
BMI: Body mass index
CBZ: Carbamazepine
IUGR: Intrauterine growth restriction
LEI: Latex Enhanced Immunoterbidimetric
LMIC: Lower and Middle-Income Countries
LSCS: Lower segment cesarean section
MCDK: Multicystic dysplastic kidneys
NVD: Normal vaginal deliveries
OFC: Occipitofrontal circumference
RLS: Resource-limited setting
RR: Relative risk
T1: First trimester
T2: Second trimester
T3: Third trimester
VPA: Sodium valproate
WWE: Women with epilepsy
